# Phylogenetic characterization of canine distemper virus from stray dogs in Kathmandu Valley

**DOI:** 10.1186/s12985-023-02071-6

**Published:** 2023-06-06

**Authors:** Prajwol Manandhar, Rajindra Napit, Saman M Pradhan, Pragun G Rajbhandari, Jessie A Moravek, Pranav R Joshi, Rima D Shrestha, Dibesh Karmacharya

**Affiliations:** 1 Center for Molecular Dynamics Nepal, Thapathali, Kathmandu Nepal; 2 BIOVAC Nepal, Banepa, Kavre, Nepal; 3 Vet for Your Pet Animal Hospital, Gapali, Bhaktapur, Nepal; 4 Kathmandu Animal Hospital and Research Centre, Thapathali, Kathmandu Nepal

**Keywords:** Canine distemper virus, Domestic dogs, Wild carnivores, Disease spillover, One health

## Abstract

Canine distemper is a highly contagious, often fatal disease caused by canine distemper virus (CDV) in domestic dogs and wild carnivores. The virus has caused mass epidemics in both wild and captive carnivores of high conservation value such as tigers, lions and leopards. Hence, understanding and managing CDV outbreaks is particularly important in Nepal, which is home to many species of threatened wild carnivores including tigers, leopards, snow leopards, dholes and wolves, and also contains a large population of stray dogs. Previous studies have suggested that CDV may pose a threat to wild carnivores, but there have not been any studies characterizing the genetic strains of the virus circulating in Nepal’s carnivores. We collected invasive and non-invasive biological samples from stray dogs in Kathmandu Valley and genetically characterized the strains of CDV in the dogs to belong to the Asia-5 lineage by using phylogenetic analysis. The same lineage also contained CDV strains sequenced from dogs, civets, red panda and lions in India. Based on our phylogenetic analysis, we think it is likely that CDV is maintained through sylvatic cycle among sympatric carnivores allowing the recurring spillovers and outbreaks. It is crucial to prevent the virus transmission from reservoir hosts to other species, especially threatened populations of large carnivores in Nepal. Hence, we recommend for regular surveillance of CDV targeting wild carnivores in addition to the domestic dogs.

**Supplementary Information:**

The online version contains supplementary material available at 10.1186/s12985-023-02071-6.

## Introduction

Canine distemper is a highly contagious, often fatal disease caused by canine distemper virus (CDV). CDV is a single-stranded enveloped RNA virus belonging to the *Morbillivirus* genus of the Paramyxoviridae family [1]. The disease is transmitted by aerosol and causes characteristic respiratory, gastrointestinal and nervous symptoms in infected domestic dogs (*Canis lupus familiaris*) and wild carnivores [1–3]. While often considered to be a dog disease, CDV has been reported in almost all members of the Carnivora order, as well as in some primates and ungulates. Importantly, CDV has been known to cause mass epidemics in wild carnivores of high conservation value. For example, CDV outbreaks have caused mass mortality of African lions (*Panthera leo leo*) in Serengeti National Park and Asiatic lions (*Panthera leo persica*) in Gir National Park [4–6]. Similarly, CDV has been isolated from deceased Amur tigers (*Panthera tigris altaica*) and Amur leopards (*Panthera pardus orientalis*) in Eastern Russia [7, 8]. The disease has also been found to infect captive tigers and leopards in zoos in the United States, as well as farmed animals like minks, ferrets, and martens in the United States and Europe [9, 10]. In recent years, these outbreaks have indicated that emerging lineages of CDV have expanded the host range of the disease [11].

Understanding and managing CDV outbreaks is particularly important in Nepal, which is home to many species of threatened wild carnivores including tigers, leopards, snow leopards (*Panthera uncia*), dholes (*Cuon alpinus*) and wolves (*Canis lupus chanco*), and also has a large population of stray dogs [12]. Moreover, a recent serosurveillance study on wild felids in Nepal confirmed CDV exposure on tigers and leopards, where more than one third of the tested big cats were symptomatic and three died soon after being sampled [13]. Similarly, previous studies have shown that CDV is prevalent in stray dog populations across Nepal. Two independent surveys on stray dog populations around Chitwan National Park and its buffer zones conducted during 2017 and 2019 identified 17% and 80% seroprevalence of CDV antibodies respectively [14, 15]. In 2018, a similar study in Manang identified CDV antibodies in 70% of dogs, while 13% tested positive in RT-PCR screening [16]. In all situations, because the dogs and wild carnivores had never been vaccinated for CDV, the presence of CDV antibodies indicated that these animals had been exposed to and/or infected with the virus. Furthermore, these incidences have been occurring in or near important Nepali wildlife regions, and the proximity of infected dogs and wild carnivore populations creates the potential for disease transmission.

Although these previous studies suggest that both dogs and wild carnivores had been exposed to CDV infections, no studies have genetically sequenced the RNA of the virus. To better understand the evolutionary origin of CDV in Nepal, we genetically characterized CDV from outbreaks in stray dogs in the Kathmandu Valley in 2018. We also speculated the possible ongoing sylvatic cycle of CDV circulation among carnivores, based on phylogenetic analysis of CDV lineages across diverse hosts by analyzing publicly available sequences from domestic and wild CDV hosts around the world.

## Materials and methods

### Study design and study site

**Fig. 1:**
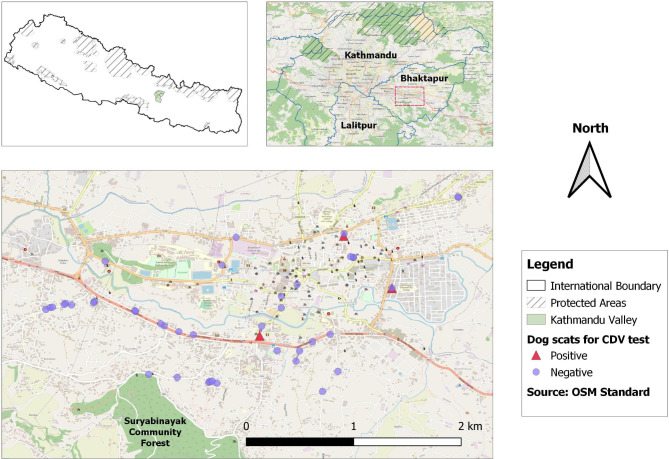
The locality in the center of Bhaktapur district, where dog scat samples were collected for detecting and characterizing CDV strains in stray dogs of Kathmandu Valley

We conducted this study to screen stray dogs for CDV in the Kathmandu Valley (Fig. 1). We chose a densely populated locality in the Bhaktapur district that is adjoined by the Suryabinayak community forest on south (Fig. 1). The Suryabinayak community forest is a contiguous forest patch in the southern outskirts of the Kathmandu Valley, and many wild carnivores like leopards, civets, martens and small felids can be found to occur in the forested hills [17].

### Sample collection

We partnered with local veterinary clinics in Kathmandu and Bhaktapur to collect invasive biological samples from stray dogs in our sampling area during early 2018 (Table S1). Local veterinary practitioners provided archived samples (ocular, saliva, fecal or rectal swabs) that had been stored frozen from stray dogs suspected to have CDV infections. The dogs demonstrated clinical symptoms of canine distemper, including conjuctivitis, hard pad, nasal and ocular discharge, seizures or bodily twitching [eg. in 18].

We also collected non-invasive fecal samples from stray dogs in central Bhaktapur (Fig. 1). We sampled the dog feces in a dense settlement area in central Bhaktapur and used a stratified sampling scheme to collect a proportional number of samples from all parts of the region. We sampled fresh dog scats in and along streets between 7 and 9 AM before the municipality cleaned the streets. The sample collector wore gloves and a facemask to prevent sample contamination and changed gloves between each sampling. We used a sterile cotton swab to dab the outer mucus layer of the scat and swirled in a cryovial filled with 0.5ml of Trizol stabilizer. We stored the samples in a -80^o^C freezer in Intrepid Nepal lab until further processing.

### Molecular screening of CDV by RT-PCR

We vortexed the samples (clinical and fecal swabs) to homogenously distribute cells in the Trizol suspension. We performed RNA extraction using the Direct-zol RNA MiniPrep kit (Zymo Research, USA). We carried out cDNA synthesis from RNA elute using Invitrogen Superscript III (ThermoFisher, USA) and stored in -80 °C freezer until downstream processing.

We performed PCR screening for the CDV *phosphoprotein* (P) gene using a *Morbillivirus* specific primer set that amplifies the ~ 390 bp region of the gene [19]. PCR amplicons were visualized on 1.5% agarose gels, and the amplified products were purified using ExoSAP-IT™ kit (Thermofisher, USA), and the sequencing reactions were performed in an MJ Research PTC-225 Peltier Thermal Cycler using the ABI PRISM® BigDye™ Terminator V3.1 Cycle Sequencing Kits (Applied Biosystems, USA) following the manufacturer’s protocols. Finally, the sequencing products were resolved on ABI 310 Genetic Analyzer (Applied Biosystems, USA), and the P gene sequences were used to confirm the presence of CDV using blast tool against Genbank database.

### Phylogenetic analysis of CDV strains

After confirming presence of CDV in samples using the P gene, we amplified a portion of the *hemagglutinin* (H) gene (approximately 852 bp) using a tiled amplicon method [20] with primers 3 F/3R and 4 F/4R, and sequenced for variant characterization in positive samples. The H gene encodes the viral surface glycoprotein that mediates attachment between the virus and the host cells. This gene has higher genetic variability than other portions of the genome and is therefore used as marker for lineage classification of CDV in most studies [21].

To characterize the lineage of the CDV strains from stray dogs in Kathmandu, we compared the sample sequences collected for our study with all currently known lineages of CDV. We compiled a dataset of 245 full-length CDV H-gene sequences (hereafter, referred as “reference sequences”) originating from at least 26 different countries across five continents (except Australia and Antarctica) collected from 1940 to 2018 (Table S2). This dataset also includes vaccine strain sequences and other sequences from 17 different geographically defined lineages of CDV from host species belonging to the Canidae, Felidae, Mustelidae, Ailuridae, Procyonidae, and Ursidae families of carnivores, which are publicly available in the NCBI GenBank database. We aligned all the reference sequences along with Kathmandu’s dog CDV sequences using MUSCLE ver3.8.425 [22], and visually inspected alignment and then trimmed and edited the sequences wherever required using AliView ver1.26 [23]. For the phylogenetic tree reconstruction, we selected the best-fit nucleotide substitution model (TVM + G) based on Bayesian Information Criterion in jModeltest2 ver2.1.8 [24] from the alignment dataset. We conducted phylogenetic analysis using this model and the Bayesian inference method in MrBayes v3.2.7 [25] with 2,000,000 iterations, sampling every 2,000 iterations and discarding the first 25% as a burn-in. We visualized and annotated the phylogenetic tree using FigTree v1.4.4 [26].

## Results

### Molecular detection

We obtained clinical samples from eight symptomatic dogs admitted at two veterinary clinics in Bhaktapur and Kathmandu districts. From the eight dogs, we obtained a total of 15 samples to test for CDV, including ocular (n = 7), rectal (n = 7) and saliva (n = 1) samples. Seven of the dogs tested positive for CDV via one or more of these sample types, while one individual dog tested negative in both ocular and rectal samples. Of the fifteen total samples taken, four ocular and four rectal samples tested positive. We also collected 44 scat samples from the streets of Bhaktapur, among which only three samples (6.8%) tested positive for CDV via *P-gene* PCR screening (Fig. 1).

### Lineage characterization and hosts diversity of CDV

**Fig. 2:**
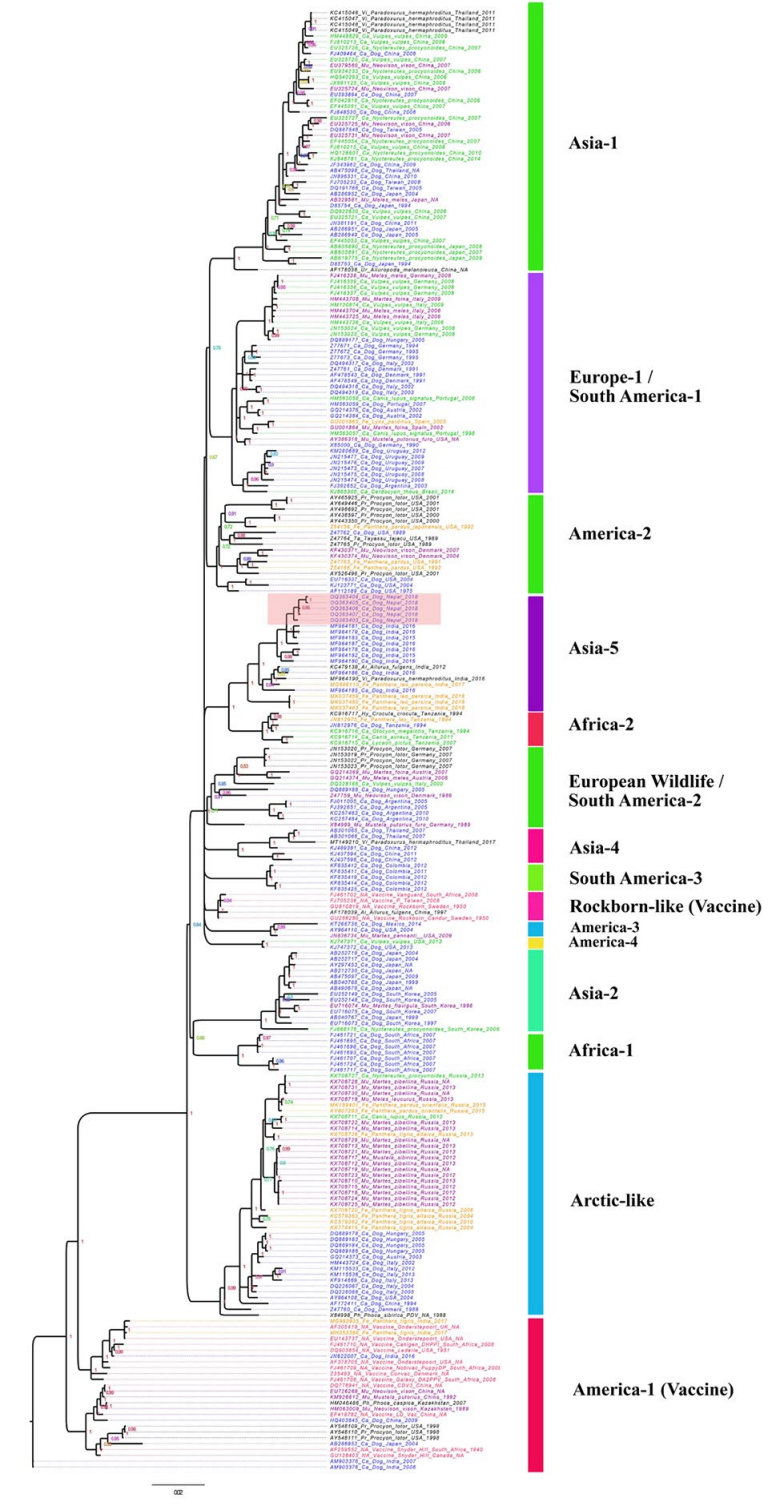
Phylogenetic tree constructed based on the 245 CDV hemagglutinin (H) gene sequences from 27 countries and 17 lineages, including the five samples collected from dogs (*Canis lupus familiaris)* in Nepal, this clade is highlighted in light red. Phylogenetic analysis was conducted using the Bayesian inference method in MrBayes with a run of 2,000,000 iterations, sub sampling per 2,000 iterations and discarding 25% of sample for burn-in. The tips of the phylogenetic tree are colored based on the family of their host species/origin. Common CDV vaccines were highlighted in red, Canidae except dogs were highlighted in green, dogs were in blue, Mustelidae in purple, Felidae in orange and all remaining were in black color. Posterior probability values of nodes estimated by Bayesian inference are indicated on the tree

We determined *H-gene* partial sequences (~ 800 bp) from five CDV samples (four clinical and one scat source) isolated from four dogs and a dog feces in the Kathmandu Valley (Table S1). From the phylogenetic analysis, the CDV strains from dogs in Kathmandu Valley were classified as Asia-5 lineage (Fig. 2). This lineage also contained CDV strains sequenced from dogs, palm civet (*Paradoxurus hermaphroditus*), red panda (*Ailurus fulgens*) and Asiatic lions in India.

We found that CDV lineages were mainly clustered according to geographic regions. Each lineage consisted of strains isolated from diverse carnivore species co-occurring in the respective geographic regions. Virus strains isolated from carnivores belonging to Canidae and Mustelidae were the most commonly occurring ones across the lineages. The Asia-5 lineage where our Kathmandu Valley dog samples clustered is a sister clade to Africa-2 lineage that consisted of strains isolated from carnivores in the Serengeti landscape including African lions, spotted hyaena (*Crocuta crocuta*), African wild dogs (*Lycaon pictus*), bat-eared fox (*Otocyon megalotis*), jackals (*Canis aureus*) and domestic dogs (Fig. 2).

## Discussion

### Phylogenetic implications of CDV in dogs and wild carnivores

Our study genetically characterized CDV strains in dogs of Kathmandu Valley as the Asia-5 lineage, which was first identified in 2016, described by Bhatt et al. (2019) [2] from dogs in India. Later, the same lineage was also found to be the causative variant behind fatal outbreaks in Asiatic lions in Gir National Park, Gujarat of western India during 2018, which caused the death of nearly two dozens of lions [6]. The same strains were also identified in Asian palm civet and red panda elsewhere in India [6, 27]. The fact that the CDV strains found in Kathmandu’s dogs are the same as those found among carnivores in India suggests that the Asia-5 variant may be prevalent among reservoir hosts in the Indian subcontinent region, specifically in India and Nepal. Similar regional patterns have been identified in other CDV strains in other regions (visualized in Fig. 2). For example, the Africa-2 variant was first identified to be responsible for death of over 1,000 African lions in Serengeti National Park, Tanzania in 1985 [4]. The same strain was later isolated from feral dogs, spotted hyaena and bat-eared fox during investigation of the outbreak, and it was again detected in African wild dogs and jackals decades later [28]. Similarly, the Arctic-like variant of CDV that was isolated from deceased Amur tigers and Amur leopards in Siberia were also found among mustelids across the region during a long-term CDV surveillance study of wild carnivores in Russia [29].

These studies suggest that CDV strains that are similar or closely related circulate regionally among sympatric carnivore hosts. This leads us to believe that CDV in wild carnivores, such as leopards and tigers, affected by the virus in Nepal [13] may also belong to the Asia-5 lineage. According to Bodgener et al. (2023), higher levels of dog predation behavior among leopards than tigers may have increased the level of associated exposure to CDV in leopards [13]. However, it is possible that dogs are not the sole contributors and that they are just playing a small role in a more complex reservoir community. In Russia, it was discovered that small wild carnivores, such as mustelids, were the most likely source of infection in big cats. In Tanzania, it was also determined that dogs were not the direct source of infection during the outbreak in the lion populations. Nepal is currently undergoing rapid phases of development, and as a result, urbanization and anthropogenic encroachment of wildlife habitats are closing gaps between forests and urban spaces. It is known that there is a high prevalence of CDV among dogs in important wildlife areas like Manang, Chitwan, and recently also among leopards and tigers close to human spaces. These findings warrant the future investigations to see if there is any spillover dynamics of CDV among domestic and wild carnivores.

As observed from our CDV phylogenetic tree (Fig. 2), in most lineages, domestic dogs are the most common CDV host or reservoir. This is likely due in part to the fact that the dog is the most easily observed/tested. Studies have suggested that CDV is mainly maintained among a group of wild carnivores, possibly small carnivore guilds such as martens, civets or mongooses that are relatively resilient in nature [29, 30], which later transmits to stray dogs from their interactions that occur when stray dogs attack or prey on the small carnivore species. This is also supported in our phylogenetic tree, where the second most common reservoir host among all lineages were mustelids. The species from Mustelidae family in our phylogenetic dataset consisted of European badger (*Meles meles*), ferret (*Mustela putorius furo*), American mink (*Neovison vison*), yellow-throated marten (*Martes flavigula*), beech marten (*Martes foina*), fisher (*Martes pennanti*), European polecat (*Mustela putorius*), sable (*Martes zibellina*), Siberian weasel (*Mustela sibirica*) and Asian badger (*Meles leucurus*) (Table S2). These mustelids are distributed mainly across Asia, Europe and North America. A comprehensive study by Gilbert et al. (2020) [29] suggested that mustelids in the Siberian wilderness may be maintaining the CDV at local level via sylvatic cycles across multi-host carnivore communities.

### Conservation implications

In countries with large stray dog populations like Nepal, dogs have the potential to become CDV vectors as they frequently interact (attack/kill) with small carnivores like mustelids, and then fall prey to large predators. These ecological dynamics increase the chances of CDV spillovers and infection from small carnivores to stray dogs to large carnivore species or vice-versa, which are already threatened by a variety of anthropogenic factors [31]. The Kathmandu Valley is one such example where CDV spillover dynamics may be actively ongoing as rapid urbanization and anthropogenic encroachment of wildlife habitats are closing the gaps between forests and urban spaces. An adult male leopard died of clinical signs consistent with CDV infection in 2021 in western outskirt region of Kathmandu [13]. Previous studies that have proven CDV exposure to dogs, leopards and tigers in different parts of Nepal suggest that this cycle may already be an ongoing process in most regions across the country. As such, more surveillance studies using molecular sequencing are required to confirm and characterize the CDV among suspected sick dogs and wild carnivores in Nepal. In addition to the carnivores, species such as ungulates and monkeys have been infected with CDV in the past and should also be surveilled [11].

Most of the surveillance efforts have focused on dogs, but it is essential for future surveillance programs to expand into wild carnivores, including mustelids, viverrids, as well as large felids and canids. Even if dogs do not have a direct role in the CDV reservoir, the unmanaged and growing populations of stray dogs can pose threats to wildlife through predation, disease transmission, and other means. Therefore, it is crucial to implement neutering and vaccination programs for dogs to address the challenges facing wildlife conservation.

## Electronic supplementary material

Below is the link to the electronic supplementary material.

Supplementary Material 1

Supplementary Material 2

## Data Availability

All the data are included within the manuscript. The nucleotide sequences generated in this study are deposited in NCBI Genbank and accessions listed in Supplementary Table S1.
