# Supplementary material for: Phylogenetic characterization of canine distemper virus from stray dogs in Kathmandu Valley
Source: Virol J. 2023 Jun 6;20:117. doi: 10.1186/s12985-023-02071-6 (PMC10245400; doi:10.1186/s12985-023-02071-6)
Supplement: Supplementary file 1 — Supplementary Material 1 [file 12985_2023_2071_MOESM1_ESM.docx]

Table S1. List of hemagglutinin gene sequences of CDV generated in this study along with their details and Genbank accession numbers.

| **S.N.** | **Sample ID** | **Sample type** | **Sample source** | **Country** | **District** | **Collection date** | **Genbank accession** |
| --- | --- | --- | --- | --- | --- | --- | --- |
| 1 | D02EST-CD3 | Clinical | occular/rectal | Nepal | Bhaktapur | 2018-01-20 | OQ363404 |
| 2 | D04EST-CD4 | Clinical | occular/rectal | Nepal | Bhaktapur | 2018-01-20 | OQ363406 |
| 3 | D06EST-CD7 | Clinical | occular | Nepal | Bhaktapur | 2018-01-20 | OQ363407 |
| 4 | D09FED-CD3 | Feces | feces | Nepal | Bhaktapur | 2018-03-27 | OQ363405 |
| 5 | D17RST-LAL | Clinical | ocular/rectal/saliva | Nepal | Kathmandu | 2018-04-03 | OQ363403 |
